# Characteristic-Grouped Adiposity Indicators for Identifying Metabolic Syndrome in Adolescents: Develop and Valid Risk Screening Tools Using Dual Population

**DOI:** 10.3390/nu12103165

**Published:** 2020-10-16

**Authors:** Yu-Ting Chin, Wei-Ting Lin, Pei-Wen Wu, Sharon Tsai, Chun-Ying Lee, David W. Seal, Ted Chen, Hsiao-Ling Huang, Chien-Hung Lee

**Affiliations:** 1 Department of Public Health, College of Health Sciences, Kaohsiung Medical University, Kaohsiung 80708, Taiwan; kiki13336586@gmail.com (Y.-T.C.); wtlin0123@gmail.com (W.-T.L.); catstar1211@gmail.com (P.-W.W.); 2 Department of Global Community Health and Behavioral Sciences, School of Public Health and Tropical Medicine, Tulane University, New Orleans, LA 70112, USA; dseal@tulane.edu (D.W.S.); tchen@tulane.edu (T.C.); 3 Department of Laboratory Medicine, Kaohsiung Municipal Siaogang Hospital, Kaohsiung 81267, Taiwan; 870718@kmuh.org.tw; 4 School of Medicine, College of Medicine, Kaohsiung Medical University, Kaohsiung 80708, Taiwan; cying@ms19.hinet.net; 5 Department of Family Medicine, Kaohsiung Medical University Hospital, Kaohsiung Medical University, Kaohsiung 807378, Taiwan; 6 Department of Oral Hygiene, College of Dental Medicine, Kaohsiung Medical University, Kaohsiung 80708, Taiwan; hhuang@kmu.edu.tw; 7 Research Center for Environmental Medicine, Kaohsiung Medical University, Kaohsiung 80708, Taiwan; 8 Department of Medical Research, Kaohsiung Medical University Hospital, Kaohsiung Medical University, Kaohsiung 80756, Taiwan

**Keywords:** adolescent, adiposity indicators, discriminatory capability, metabolic syndrome, obesity, principal component analysis, risk screening tool, Taiwan

## Abstract

A simple, robust, and characterized adiposity indicator may be appropriate to be used as a risk screening tool for identifying metabolic syndrome (MetS) in adolescents. This study used dual adolescent populations to develop and validate efficient adiposity indicators from 12 characterized candidates for identifying MetS that may occur during the transition from adolescence to young adulthood. Data from the adolescent Nutrition and Health Survey in Taiwan (*n* = 1920, 12–18 years) and the multilevel Risk Profiles for adolescent MetS study (*n* = 2727, 12–16 years) were respectively used as training and validation datasets. The diagnostic criteria defined by the International Diabetes Federation for adolescents (IDF-adoMetS) and the Joint Interim Statement for adults (JIS-AdMetS) were employed to evaluate MetS. In the training dataset, principal component analysis converted 12 interrelated obesity indices into bodyfat-, lipid-, and body-shape-enhanced groups, with the first two characteristic-groups having a higher discriminatory capability in identifying IDF-adoMetS and JIS-AdMetS. In the validation dataset, abdominal volume index (AVI) among girls and waist circumference (WC) among boys were respectively validated to have a higher Youden’s index (0.740–0.816 and 0.798–0.884) in identifying the two MetS. Every 7.4 and 4.3 positive tests of AVI (cutoff = 13.96) had an accurate IDF-adoMetS and JIS-AdMetS, respectively, and every 32.4 total tests of WC (cutoff = 90.5 cm) had a correct identification for the two MetS. This study stresses the discriminatory capability of bodyfat- and lipid-enhanced adiposity indicators for identifying MetS. AVI and WC were, respectively, supported as a risk screening tool for identifying female and male MetS as adolescents transition to adulthood.

## 1. Introduction

Metabolic syndrome (MetS) is a health hazard condition that reflects a constellation of several cardiometabolic risk factors, including excessive abdominal adiposity, high blood pressure and fasting plasma glucose, and abnormal triglyceride and high-density lipoprotein-cholesterol [1]. Longitudinal studies have demonstrated that MetS makes adolescents higher susceptible to developing MetS, type 2 diabetes mellitus, and cardiovascular diseases in adult life [2,3,4]. Because MetS and its risk components occurring in childhood can be restored to normal [5], identifying and treating adolescents with this syndrome through an efficient risk screening implement is an advocated strategy for controlling subsequent adverse disease consequence.

Among 5 components of MetS, 3 cardiometabolic dysfunctions must be determined via blood biochemical examination. Because blood assessment is an invasive and more costly test approach, using it as a universal screening tool for MetS may be less feasible among younger children. Therefore, the development and validation of a simple and robust screening instrument for identifying adolescent MetS is a vital work for pediatric public health.

Anthropometric studies have reported that, compared with entire body fatness measured by body mass index (BMI), regional fat distribution is more associated with metabolic disturbances and cardiovascular health risks [6]. Adiposity investigations into measurement of bodyfat distribution have indicated that body adiposity index (BAI) can directly estimate bodyfat percentage [7]; body roundness index (BRI) is a predictor of the percentages of bodyfat and visceral adipose tissue [8]; waist-to-height ratio (WHtR) acts as a better risk marker for cardiovascular risk and shorter lifespan than BMI [9,10]; abdominal volume index (AVI) can be employed to estimate overall abdominal volume that theoretically comprises intra-abdominal fat and adipose tissue volumes [11]; waist circumference (WC) was a stronger predictor of obesity-related cancers [12]; waist-to-hip ratio (WHR) measures apple- or pear-like body shape [13]; a body shape index (ABSI) expresses the excess risk from high WC adjusted for the effect of BMI [14]; and conicity index (CoI) is a double cone-shaped derived indicator for abdominal obesity that can better predict 10-year cardiovascular risk [15,16]. In insulin and fat function studies, triglyceride-glucose index (TGI) was used as a surrogate for identifying insulin resistance among healthy subjects [17], and visceral adiposity index (VAI) was identified as an indicator of visceral adipose function and insulin sensitivity [18]. Furthermore, lipid accumulation product (LAP) has been recommended as a lipid overaccumulation indicator [19]. Although these adiposity indices were developed from adult populations, they may be appropriate to be used as a risk screening tool for identifying adolescent MetS, given that obesity has been a major risk for adult MetS [20,21].

Adiposity indices created to measure body adiposity, abdominal obesity, body shape, and visceral fat accumulation are a group of interrelated variables that may have a characteristic aggregation. Principal component (PC) analysis is a statistical method that can convert interrelated variables into reduced independent and interpretable PCs with a specific characteristic through multilinear subspace algorithms, and produce a characteristic-weighted combined score for each retained PC [22]. This technique has been used to study the clustering of pediatric cardiometabolic risk factors [23,24], and is an appropriate method to investigate the characteristic groups of adiposity indicators.

In one school-based longitudinal investigation of the alteration of MetS typology, 16.4%, 5.7%, and 1.1% of adolescents were observed to have new incident, unstable/remitted, or persistent MetS, respectively, as adolescents aged into young adulthood [5]. This raises the question of what kind of adiposity indicators can effectively identify MetS for children in school settings using criteria for adolescents and young adults. In the present study, data from the adolescent Nutrition and Health Survey in Taiwan (ado-NAHSIT) was used to develop efficient adiposity indicators from 12 characterized candidates for identifying MetS that may occur during the transition from adolescence to young adulthood. Data from the multilevel Risk Profiles for adolescent Metabolic Syndrome (mRP-aMS) study was employed to validate the accuracy and efficiency of determining MetS for the selected adiposity indicators.

## 2. Materials and Methods

### 2.1. Study Participants

The development and validation of adiposity indicators for this study were carried out using 2 sample series of participants. The first sample, obtained from the ado-NAHSIT, was used as a training dataset to derive optimal adiposity indicators for the identification of MetS using the criteria for adolescents and young adults. Two commonly applied diagnosis criteria for the 2 types of MetS were used. The second sample, obtained from the mRP-aMS study, was used as a validation dataset to verify the accuracy and efficiency of determining each MetS for the chosen obesity indicators. All participants and their guardians in the two studies provided a written informed assent and consent. The research protocol for this study was approved by the Institutional Review Board of Kaohsiung Medical University Hospital (IRB No., KMUHIRB-20120103; date of approval, 16 August 2019).

### 2.2. The Adolescent Nutrition and Health Survey in Taiwan (ado-NAHSIT)

The ado-NAHSIT as a nationwide adolescent survey in Taiwan that used a multistage, geographic area and population density stratified random sampling design to recruit nationally representative samples for investigating the health and nutritional status of adolescents aged 12–18 years during 2010–2011 [25]. Adolescents who had an official student status in the period of 2010–2011 and were enrolled in the public/private junior high schools and senior high/vocational schools in Taiwan were included in the study population; however, the adolescents who were enrolled in extension schools, special schools, and schools for overseas Chinese were excluded in the study population [26]. A comprehensive working group, consisting of trained interviewers, nutritionists, and medical personnel was organized to collect sociodemographic characteristics, dietary patterns, lifestyle factors, physical activity, puberty status, health and disease related information, anthropometry measurements, and clinical biochemical data from study participants. A detailed explanation for data collection, measurements, and laboratory assessments has been given previously [24,25,26,27]. Data were preserved for analysis of 1920 adolescents (971 girls and 949 boys) who had complete study variables and clinical parameters used to determine MetS (5 participants were excluded due to no information on sex variable).

### 2.3. The Multilevel Risk Profiles for Adolescent Metabolic Syndrome (mRP-aMS) Study

The mRP-aMS investigation was a large-scale cross-sectional study that employed a multistage, geographically stratified cluster sampling scheme to recruit representative adolescents for monitoring multilevel risk profiles of MetS in southern Taiwan during 2007–2009 [28,29,30,31]. In this study, data were collected about demographic characteristics, dietary patterns, lifestyle factors, physical activity, anthropometry variables, and biochemical data for 2727 adolescents (1399 girls and 1328 boys, response rate: 72.1%), aged 12–16 years [28,29]. All participants were included in the dataset for validating the accuracy of adiposity indicators in identifying MetS.

### 2.4. Diagnosis of Metabolic Syndrome

The International Diabetes Federation definition of MetS for adolescents aged 10–18 years (IDF-adoMetS) and the Joint Interim Statement for adult MetS (JIS-AdMetS) were respectively employed to determine MetS [20,32]. The 2 definitions correspond to a worldwide consensus for MetS diagnosis in adolescents and young adults. The categorical criteria and discrepancies for each risk component of IDF-adoMetS and JIS-AdMetS—including central obesity, high blood pressure, low high-density lipoprotein cholesterol, increased triglyceride, and elevated fasting plasma glucose—are presented in Supplementary Table S1. The IDF-adoMetS diagnosis requires an adolescent to have central obesity and 2 other abnormal components [32]. The JIS-AdMetS diagnosis requires having ≥3 any abnormal components [20].

### 2.5. Single Adiposity Indicators

Body weight, height, WC, and hip circumference was measured according to World Heatlh Organization standards [33]. Adiposity indicators—including BMI, BAI [7], BRI [8], WHR [13], WHtR [10], AVI [11], ABSI [14], CoI [15], TGI [17], VAI [18], and LAP [19]—were calculated using the following formulas:

$$\mathrm{BMI}=\mathrm{Weight}\;\left(\mathrm{kg}\right)/{\mathrm{Height}}^{2}\left(m\right)\;\mathrm{BAI}=\left[\mathrm{Hip}\;\mathrm{circumference}\;\left(\mathrm{cm}\right)/{\mathrm{Height}}^{1.5}\;\left(m\right)\right]-18\;\mathrm{BRI}=364.2-365.5\times {\left\{1-{\left[\frac{\mathrm{WC}\;\left(m\right)}{\pi \times \mathrm{Height}\;\left(m\right)}\right]}^{2}\right\}}^{1/2}\;\mathrm{WHR}=\mathrm{WC}\;\left(\mathrm{cm}\right)/\mathrm{Hip}\;\mathrm{circumference}\;\left(\mathrm{cm}\right)\;\mathrm{WHtR}=\mathrm{WC}\;\left(\mathrm{cm}\right)/\mathrm{Height}\;\left(\mathrm{cm}\right)\;\mathrm{AVI}=\left\{2\times {\mathrm{WC}}^{2}\;\left(\mathrm{cm}\right)+0.7\times {\left[\mathrm{WC}\;\left(\mathrm{cm}\right)-\;\mathrm{Hip}\;\mathrm{circumference}\;\left(\mathrm{cm}\right)\right]}^{2}\right\}/1000\;\mathrm{ABSI}=\mathrm{WC}\;\left(m\right)/\left[{\mathrm{BMI}}^{2/3}\times {\mathrm{Height}}^{1/2}\;\left(m\right)\right]\;\mathrm{CoI}=\mathrm{WC}\;\left(m\right)/\left[0.109\times \sqrt{\mathrm{weight}\;\left(\mathrm{kg}\right)/\mathrm{height}\;\left(m\right)}\right]\;\mathrm{TGI}=\ln\left[\mathrm{TG}\;\left(\mathrm{mg}/\mathrm{dL}\right)\times \mathrm{FPG}\;\left(\mathrm{mg}/\mathrm{dL}\right)/2\right]\;{\mathrm{VAI}}_{\mathrm{female}}=\left[\frac{\mathrm{WC}\;\left(\mathrm{cm}\right)}{36.58+\left(1.89\times \mathrm{BMI}\right)}\right]\times \left[\frac{\mathrm{Triglyceride}\;\left(\mathrm{mmol}/l\right)}{0.81}\right]\times \left[\frac{1.52}{\mathrm{HDL}\;\left(\mathrm{mmol}/l\right)}\right]\;{\mathrm{VAI}}_{\mathrm{male}}=\left[\frac{\mathrm{WC}\;\left(\mathrm{cm}\right)}{39.68+\left(1.88\times \mathrm{BMI}\right)}\right]\times \left[\frac{\mathrm{Triglyceride}\;\left(\mathrm{mmol}/l\right)}{1.03}\right]\times \left[\frac{1.31}{\mathrm{HDL}\;\left(\mathrm{mmol}/l\right)}\right]\;{\mathrm{LAP}}_{\mathrm{female}}=\left[\mathrm{WC}\;\left(\mathrm{cm}\right)-58\right]\times \mathrm{Triglyceride}\;\left(\mathrm{mmol}/L\right)\;{\mathrm{LAP}}_{\mathrm{male}}=\left[\mathrm{WC}\;\left(\mathrm{cm}\right)-65\right]\times \mathrm{Triglyceride}\;\left(\mathrm{mmol}/L\right)$$

To avoid the occurrence of non-positive values in LAP, we assigned the WC values of 59 cm and 66 cm to female and male participants, respectively, who had a negative LAP value [34]. This process does not affect the evaluation of discriminatory ability for this adiposity indicator.

### 2.6. Combined Adiposity Indicators

The indicators studied are adiposity-based variables that correlate with each other and may have a characteristic clustering. A combined score that additionally weights characterized adiposity indicators may offer a better discriminatory capability in identifying MetS. To investigate this issue, we transformed 12 adiposity indicators into 3 uncorrelated PCs that explained the majority of overall variance using PC analysis, as has been done in previous studies [22,23,35]. The technique of varimax rotation was employed to obtain 3 combined adiposity scores for the 3 PCs. Each combined score is a linear sum of the z-score of each adiposity indicator multiplying its corresponding factor loading. A factor loading represents the weight of an adiposity indicator in the linear sum and measures the correlation between an indicator and a combined score.

### 2.7. Statistical Analysis

**Figure 1:**
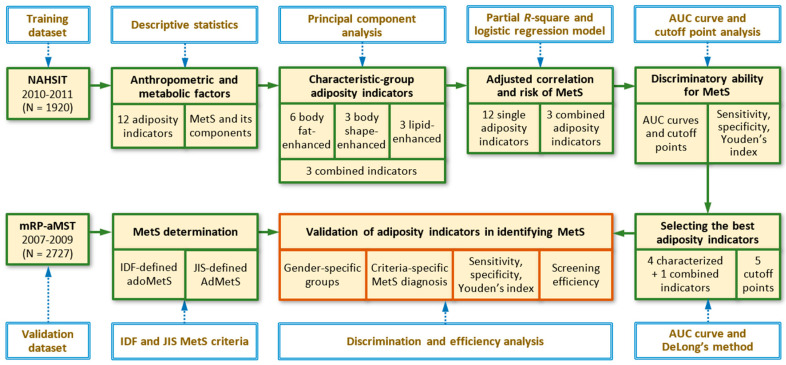
Schematic diagram of data analysis for the development and validation of adiposity indicators in discriminating adolescent metabolic syndrome. MetS, metabolic syndrome; IDF-defined adoMetS, International Diabetes Federation-defined adolescent MetS; JIS-defined AdMetS, Joint Interim Statement for adult MetS; AUC, area under receiver operating characteristic curve.

Figure 1 presents a schematic framework of 6 analytic procedures for the development and validation of adiposity indicators using the training and validation datasets. First, the distributions of anthropometric characteristics and adiposity indicators for adolescents in the ado-NAHSIT and mRP-aMS studies were analyzed using means, standard deviations, or percentages. Second, PC analysis was used to determine the retained PCs from 12 adiposity indicators according to the criteria: eigenvalues ≥1 or PCs that exceeded the break in the scree plot [22,23]. The first 3 PCs—including PC1, PC2, and PC3—were retained. Because of the clustering, the 12 obesity indices were grouped as bodyfat-, body-shape-, and lipid-enhanced adiposity indicators. Third, partial correlation (pCorr), partial *R*-square (p*R*^2^), and logistic regression-derived odds ratio (OR) were used to measure the adjusted correlation, contribution, and risk of each adiposity indicator for IDF-adoMetS and JIS-AdMetS, respectively, after controlling for possible confounding effects. Adjusted covariates included study area, age, daily energy intake, physical activity, puberty status, cigarette smoking, and alcohol drinking.

Fourth, area under receiver operating characteristic curve (AUC) and the sensitivity and specificity of each cutoff point were calculated for each adiposity indicator, and were used to evaluate the discriminatory ability in identifying IDF-adoMetS and JIS-AdMetS. The best cutoff points for each MetS identification were determined by maximizing the Youden’s index (YI, i.e., sensitivity+specificity-1). Fifth, the adiposity indicators that have the greatest AUC and/or YI in each characteristic-group and in the PC score group were selected to verify their discriminatory capability in identifying each MetS. We employed DeLong et al.’s non-parametric approach to evaluate the difference of AUCs across the selected adiposity indicators [36]. Lastly, the external discriminatory abilities for the selected indicators in identifying each MetS were verified in the validation dataset for both sexes. Sensitivity, specificity, the number of positive tests per case identified, and total number of tests per case identified were used to evaluate screening efficiency of identifying MetS. All of the data were analyzed using the statistical software Stata version 16 (StataCorp., College Station, TX, USA).

## 3. Results

Table 1 displays the distribution of anthropometric characteristics, obesity indicators, and MetS, stratified by sex, for adolescents in the ado-NAHSIT and mRP-aMS studies. In both investigations, sex differences in anthropometric parameters and adiposity indicators were notable. The boys had higher levels of WC, systolic blood pressure, and fasting plasma glucose than the girls, whereas the girls had greater levels of high-density lipoprotein cholesterol than the boys. In the ado-NAHSIT study, 2.37% and 4.11% of girls and boys, respectively, had IDF-adoMetS; and 3.30% and 4.53% of girls and boys, respectively, had JIS-AdMetS. In the mRP-aMS investigation, 1.43% and 3.16% of female and male adolescents, respectively, had IDF-adoMetS; and 2.72% and 3.46% of female and male adolescents, respectively, had JIS-AdMetS.

In the training dataset, the principal component analysis (PCA) procedure converted 12 obesity indicators into a comparable PC structure in each sex, with the first 3 PCs explaining 92.4% and 93.8% of the overall variance for girls and boys, respectively (Table 2). Among girls, PC1, PC2, and PC3 scores were respectively more correlated with bodyfat-, body-shape-, and lipid-enhanced adiposity indicators (factor loadings: 0.366–0.419, 0.416–0.656, and 0.417–0.650; total variance explained: 52.7%, 20.7%, and 19.0%, respectively). Among boys, PC1 to PC3 were separately more associated with bodyfat-, lipid-, and body-shape-enhanced obesity indicators (factor loadings: 0.365–0.414, 0.314–0.682, and 0.377–0.767; total variance explained: 59.7%, 17.8%, and 16.3%, respectively).

**Figure 2:**
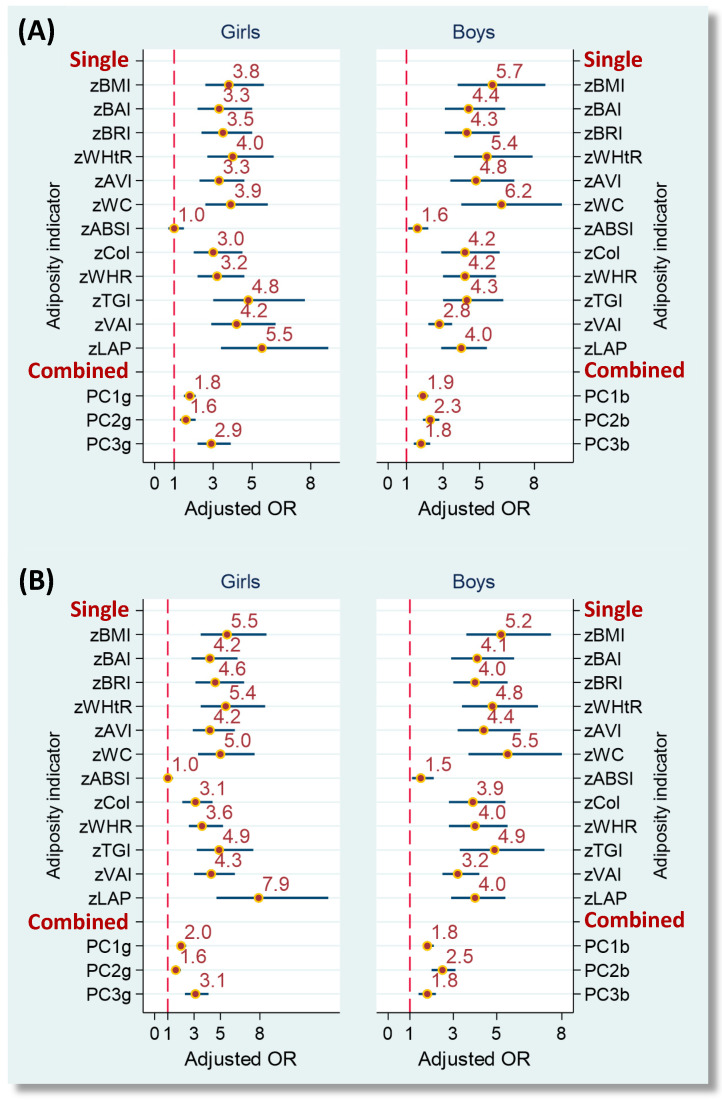
Adjusted odds ratios of IDF-adoMetS (**A**) and JIS-AdMetS (**B**) associated with single and combined adiposity indicators in adolescents, the ado-NAHSIT study. Adjusted ORs were adjusted for study area, age, daily energy intake, physical activity, puberty status, cigarette smoking and alcohol drinking. Adiposity indicators were standardized to a z-score. MetS, metabolic syndrome; OR, odds ratio; IDF-adoMetS, International Diabetes Federation-defined adolescent MetS; JIS-AdMetS, Joint Interim Statement for adult MetS; BMI, body mass index; BAI, body adiposity index; BRI, body roundness index; WHtR, waist-to-height ratio; AVI, abdominal volume index; WC, waist circumference; ABSI, a body shape index; CoI, conicity index; WHR, waist-to-hip ratio; TGI, triglyceride-glucose index; VAI, visceral adiposity index; LAP, lipid accumulation product; PC1g-PC3g, principal component 1 to 3 for girls; PC1b-PC3b, principal component 1 to 3 for boys.

Figure 2 illustrates the covariate-adjusted risks of IDF-adoMetS and JIS-AdMetS associated with single and combined adiposity indicators in the training dataset. Apart from ABSI for the girls, all obesity indicators were associated with a significantly higher risk of developing MetS. Standardized LAP was the strongest risk factor for girls in the IDF-adoMetS and JIS-AdMetS (adjusted OR = 5.5 and 7.9, respectively), while standardized WC was the strongest risk factor for boys in the 2 MetS (adjusted OR = 6.2 and 5.5, respectively).

Table 3 presents the pCorr and p*R*^2^ of single and combined adiposity indicators associated with the number of abnormal IDF-adoMetS and JIS-AdMetS components in ado-NAHSIT participants. For the IDF-adoMetS, AVI, WHR, LAP, and PC1 respectively had the highest pCorr (0.613, 0.443, 0.613, and 0.621 in girls and 0.623, 0.502, 0.648, and 0.622 in boys) in bodyfat-enhanced, body-shape-enhanced, lipid-enhanced, and combined adiposity index groups, after adjusting for the covariates. These obesity indicators separately explained the greatest variability in the number of abnormal IDF-adoMetS components in each group (p*R*^2^, 19.6–38.6% in girls; 25.2–42.1% in boys). Comparable pCorr and p*R*^2^ patterns for these adiposity indicators were observed for the JIS-AdMetS.

Table 4 presents the discriminatory abilities of adiposity indicators in identification of IDF-adoMetS and JIS-AdMetS among ado-NAHSIT adolescents. For both sexes, AVI/WC, WHR, LAP, and PC1 respectively had the greatest AUC for identifying IDF-adoMetS (0.941/0.941, 0.826, 0.942, and 0.939 among girls; 0.955/0.955, 0.898, 0.956, and 0.953 among boys) in bodyfat-enhanced, body- shape-enhanced, lipid-enhanced, and combined indicator groups. Similar results for these 5 indicators were found for the JIS-AdMetS (0.916/0.916, 0.833, 0.921, and 0.918 among girls; 0.922/0.922, 0.871, 0.938, and 0.922 among boys). YI provided comparable information for the two MetS. Using the AVI cutoff points of 13.96 for girls and 16.57 for boys, respectively, this adiposity indicator revealed a superior discrimination in identifying IDF-adoMetS (sensitivity/specificity: 95.7%/86.7% among girls; 100.0%/88.4% among boys) and JIS-AdMetS (sensitivity/specificity: 93.8%/87.4% among girls and 90.7%/88.3% among boys).

**Figure 3:**
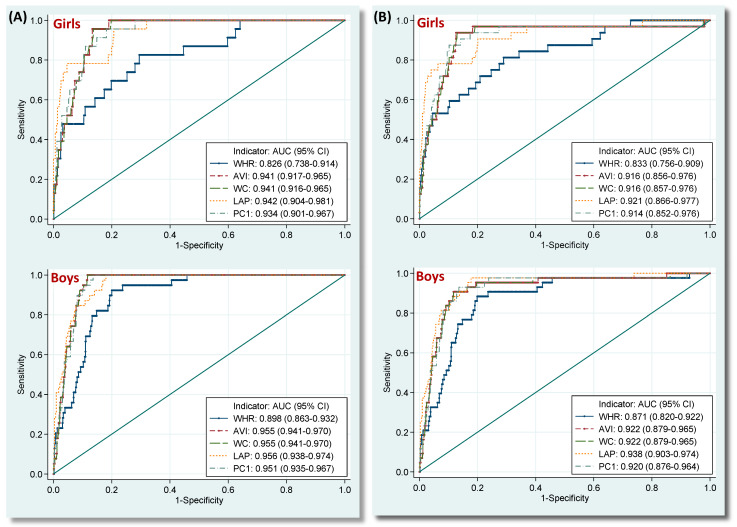
Receiver operating characteristic curves of identifying (**A**) IDF-adoMetS and (**B**) JIS-AdMetS for selected adiposity indicators in girls and boys, the ado-NAHSIT study. The AUCs of identifying IDF-adoMetS for AVI, WC, LAP, and PC1 were significantly higher than that for WHR in girls and boys (all *p* values ≤ 0.020), and the AUCs of determining JIS-AdMetS for AVI, WC, LAP, and PC1 were also significantly greater than that for WHR in girls and boys (all *p* values ≤ 0.028). AUC, area under receiver operating characteristic curve; WHR, waist-to-hip ratio; AVI, abdominal volume index; WC, waist circumference; LAP, lipid accumulation product; PC1, principal component 1 (bodyfat-enhanced factor).

Figure 3 illustrates the differences in AUCs for identifying IDF-adoMetS and JIS-AdMetS across 5 superlative adiposity indicators in the training dataset. The AUCs were compatible across AVI, WC, LAP, and PC1. Nevertheless, all were significantly greater than that for WHR for the 2 MetS across both sexes (all *p*-values ≤ 0.028).

Table 5 presents the MetS discriminatory ability for the selected adiposity indicators in mRP-aMS adolescents using the cutoff points determined by the training dataset. Among girls, AVI (0.816) and PC1 (0.826) had the highest YI for identifying IDF-adoMetS and JIS-AdMetS, respectively, and the second highest YI was WC (0.814) and AVI (0.740). Among boys, WC provided the greatest YI for IDF-adoMetS (0.884) and JIS-AdMetS (0.798), and AVI (0.860) and PC1 (0.787) offered the second highest YI for the 2 MetS, respectively. For girls, AVI had a superior identification efficiency in positive test number, in that every 7.4 and 4.3 positive tests of AVI had a correct IDF-adoMetS and JIS-AdMetS identification. For boys, WC had an exceptional detection efficiency in total test number, in that every 32.4 tests of WC had an accurate identification in both MetS.

## 4. Discussion

Except for the ABSI for girls, the single and combined adiposity indicators investigated were all qualified as a risk marker for IDF-adoMetS and JIS-AdMetS after taking multiple confounding effects into account. In the training dataset, AVI/WC, WHR, LAP, and PC1 were, respectively, the principal adiposity indicators for identifying the 2 MetS among bodyfat-enhanced, body-shape-enhanced, lipid-enhanced, and combined indicator groups for both sexes. In the validation dataset, AVI/WC for IDF-adoMetS and PC1/AVI for JIS-AdMetS among girls, and WC/AVI for IDF-adoMetS and WC/PC1 for JIS-AdMetS among boys were validated to have an excellent discrimination for the identification of MetS.

For the diagnosis of MetS, the IDF-adoMetS criteria (central obesity + 2 other abnormal components) are stricter than that for JIS-AdMetS (any 3 or more abnormal components) [20,32]; thus, the proportions of MetS for IDF-adoMetS in the 2 study samples were both lower than that for JIS-AdMetS (Table 1). Adolescence is an important growth stage that can carry health hazards into young adulthood. Our study examined and validated the accuracy of adiposity indicators for determining IDF-adoMetS and JIS-AdMetS. This method can investigate the possible application for obesity indicators in identifying MetS during the transition from adolescence to young adulthood.

The assessed obesity indicators were derived from mathematical models for abdominal fat and adipose tissue volumes [11,13,14,15], the measurements of bodyfat and visceral adipose tissue using dual energy X-ray absorptiometry and/or magnetic resonance imaging [7,8], or their association with visceral fat dysfunction, central lipid accumulation, and cardiometabolic risks [9,10,17,18,19,37]. Nevertheless, their correlations with each other are undiscussed. Our PC analysis indicated that BMI, BAI, BRI, WHtR, AVI, and WC were relatedly clustered in bodyfat-enhanced PC, ABSI, CoI, and WHR in body shape-enhanced PC, and TGI, VAI and LAP in lipid-enhanced PC, respectively. This implies that these adiposity indicators have characteristic aggregation. Additionally, the 3 characterized PC scores were associated with IDF-adoMetS and JIS-AdMetS in a different risk (lipid-enhanced PC score having the highest risk in both MetS, Figure 2) and with abnormal MetS component numbers in a heterogenous correlation (bodyfat-enhanced PC score having the greatest correlation, Table 3) among both sexes. These findings suggest that characteristic-specific adiposity indicators should be separately considered in the valuation of their associations with various health risks given their interrelated nature.

This study revealed that all adiposity indicators (ABSI aside) were positively associated with IDF-adoMetS and JIS-AdMetS risks and their abnormal component numbers among girls and boys, and the associations were statistically independent of potential confounders. Obesity is a vital contributor of adolescent MetS [21,32]. In a meta-analysis, childhood adiposity was verified as a significant predictor for the risks of abnormal carotid intima media thickness and dysglycemia in adulthood [38]. Although specific nutritional intake and lifestyles might be valuable for enhancing the accuracy of MetS screening, the adiposity indictor is still a single, simple, robust, and applicable risk-screening tool for identifying adolescent MetS in the community. Even the selection of an appropriate indicator and its associated cutoff point may depend on sex, age, and ethnic/cultural group. ABSI is an indicator that was created to adjust for the effect of BMI [14]. As observed in this study this index was not substantially associated with the BMI-correlated MetS outcomes.

In the training dataset, we found that bodyfat- and lipid-enhanced adiposity indicators generally had a better discriminating ability for identifying IDF-adoMetS and JIS-AdMetS than did body-shape-enhanced indicators among both sexes (AUCs, 0.830–0.956 vs. 0.489–0.898, Table 4). The AUCs for the superlative adiposity indicators of bodyfat- and lipid-enhanced groups were both significantly higher than that for the body-shape-enhanced group (Figure 3). Brazilian, Chilean, and Spanish investigators have recommended the bodyfat-enhanced indicators WHtR, AVI, and BMI as an excellent screening tool for adolescent MetS [39,40,41,42]. Although no adolescent studies have investigated LAP and VAI, one adult study reported that LAP was the most accurate indicator for determining male and female MetS [43]. Obesity is a growing global health problem that increases the risk of multiple physical and mental disorders [44,45,46]. These results indicate that bodyfat- and lipid-enhanced adiposity indicators should be intensively applied in risk assessments of the association between pediatric obesity and cardiometabolic diseases.

Studies that have evaluated anthropometric indicators for the identification of adolescent MetS in the community have found that AVI and WC for both girls and boys in Spain, BMI/WC for females and WHtR/WC for males in Chile, and WHtR for both sexes in Brazil have an excellent capacity for discriminating MetS [39,40,41]. However, there was a lack of validation of screening accuracy and efficiency for the indicators in these studies. Using a comparable adolescent population for validation, this investigation demonstrated that AVI/WC for girls and WC/AVI for boys had an excellent discriminating ability for identifying IDF-adoMetS in the community; however, the best indicators in discriminating JIS-AdMetS were PC1/AVI for girls and WC/PC1 for boys. This indicates that the AVI- and WC-included bodyfat-enhanced combined score (PC1) becomes more significant in detecting JIS-AdMetS. Although PC1 was recognized as an excellent adiposity indicator for identifying JIS-AdMetS, it requires intricate calculation. In community practices, our study suggests AVI as a female, and WC as a male, risk screening tool for MetS that can be applied to the transition from adolescence to young adulthood in a Taiwanese population.

In this study, the AVI cutoff points for identifying female IDF-adoMetS and JIS-AdMetS were both 13.96. Evaluated in the validation sample, 90.0% and 81.6% of sensitivity and 91.6% and 92.4% of specificity were observed (YI, 0.816 and 0.740), respectively, for the two MetS. AVI also was recommended as a risk-screening instrument for identifying female IDF-adoMetS in Spanish adolescents; however, the YI of this indicator with cutoff of 10.89 was only 0.70 (sensitivity, 100.0% and specificity, 70.0%) [39]. In our investigation, the WC cutoff points for determining male IDF-adoMetS and JIS-AdMetS were both 90.5 cm, which is close to the abnormal level of central obesity defined for Asian adults (WC ≥ 90 cm) [20]. Evaluated in the validation sample, every 32.4 tests of WC were observed to have an accurate IDF-adoMetS and JIS-AdMetS, respectively. In Spain, WC was suggested as an anthropometric discriminator for identifying male IDF-adoMetS, in which the cutoff of 75.0 cm rendered a 0.57 of YI (sensitivity, 100.0% and specificity, 75.0%) [39]. The discriminating abilities of AVI and WC for identifying IDF-adoMetS were confirmed in a southeast Spanish adolescent population [47].

In a clinically established adolescent database, if the blood samples of the participants had not been collected, or blood samples had not been examined for MetS-related variables, an adiposity indicator with very high sensitivity may be used as a tool for discovering adolescent MetS. Furthermore, an adiposity indicator with very high specificity may be employed as an instrument for confirming adolescent MetS. This framework can be applied to clinical settings. However, the appropriate adiposity indicators and their associated cutoff points have to be developed for the study population.

This study had several strengths. First, a large-scale nationally representative sample was used to develop efficient adiposity indicators and the accuracy and efficiency of the selected indicators were validated in another large-scale representative sample. Second, the clustering characteristics of obesity indicators and characterized functions were evaluated and validated. Third, multiple adiposity indicators were concurrently assessed and were used to investigate their discriminating capability for identifying MetS in the transition from adolescence to young adulthood. Fourth, although appropriate adiposity indicators and their cutoff points might vary by study populations, our research methodology and network can be applied to other countries that want to develop their specific obesity indicators. Alternatively, we recognize the limitations of our analyses. First, the recommended adiposity indicators had no causally predictive capability in determining MetS since they were all developed in a cross-sectional nature. Second, the participants used to develop indicators were Taiwanese adolescents, thus our findings may not be directly generalizable to other populations.

## 5. Conclusions

In cardiometabology, this study uncovered the characteristic cluster of adiposity indicators. Bodyfat- and lipid-enhanced adiposity indicators revealed a higher risk and discriminatory ability for MetS than did body-shape-enhanced indicators in adolescents. This highlights the consideration of indicator characteristics when evaluating the association between pediatric obesity and cardiometabolic diseases. In public health, the findings from the development and validation procedures support AVI as a female, and WC as a male, risk-screening tool for MetS that can be applied during the transition from adolescence to young adulthood in Taiwan.

## Figures and Tables

**Table 1:** Distributions of anthropometric characteristics, obesity indicators, and metabolic syndrome (MetS) for adolescents in the adolescent Nutrition and Health Survey in Taiwan (ado-NAHSIT) and multilevel Risk Profiles for adolescent Metabolic Syndrome (mRP-aMS) studies.

	ado-NAHSIT			mRP-aMS		
Variables ^1^	Girls	Boys	*p* ^2^	Girls	Boys	*p* ^2^
	(*n* = 971)	(*n* = 949)	Value	(*n* = 1399)	(*n* = 1328)	Value
Age, year	15.13 ± 1.86	15.18 ± 1.85	0.593	13.43 ± 1.02	13.43 ± 1.04	0.934
Weight, Kg	52.60 ± 10.50	62.39 ± 15.64	<0.001	50.92 ± 11.39	57.97 ± 15.84	<0.001
Height, cm	158.18 ± 5.81	168.02 ± 8.37	<0.001	156.20 ± 5.96	162.03 ± 8.85	<0.001
Hip circumference, cm	93.56 ± 7.81	94.13 ± 10.14	0.208	89.98 ± 8.45	91.16 ± 10.52	0.001
**Adiposity indicators**						
Body mass index	20.97 ± 3.68	21.96 ± 4.71	<0.001	20.79 ± 4.06	21.88 ± 4.92	<0.001
Body adiposity index	29.07 ± 3.79	25.24 ± 4.10	<0.001	28.11 ± 3.98	26.23 ± 4.47	<0.001
Body roundness index	2.97 ± 1.01	2.76 ± 1.28	<0.001	2.46 ± 1.06	2.71 ± 1.32	<0.001
Waist-to-hip ratio	0.80 ± 0.05	0.82 ± 0.06	<0.001	0.77 ± 0.06	0.81 ± 0.07	<0.001
Waist-to-height ratio	0.48 ± 0.05	0.46 ± 0.07	<0.001	0.45 ± 0.06	0.46 ± 0.07	<0.001
Abdominal volume index	11.72 ± 2.80	12.54 ± 3.99	<0.001	10.18 ± 2.88	11.57 ± 3.92	<0.001
A body shape index, 10^−1^	0.79 ± 0.03	0.77 ± 0.03	<0.001	0.74 ± 0.05	0.75 ± 0.05	<0.001
Conicity index	1.20 ± 0.05	1.17 ± 0.06	<0.001	1.12 ± 0.08	1.15 ± 0.08	<0.001
Triglyceride-glucose index	8.04 ± 0.38	8.07 ± 0.42	0.054	8.03 ± 0.43	8.04 ± 0.48	0.602
Visceral adiposity index	2.52 ± 1.72	1.90 ± 1.33	<0.001	2.38 ± 1.40	1.73 ± 1.14	<0.001
Lipid accumulation product	72.14 ± 67.53	61.23 ± 78.42	0.001	53.38 ± 62.13	53.35 ± 77.21	0.986
**Components of MetS**						
Waist circumference, cm	75.23 ± 8.71	77.60 ± 12.01	<0.001	69.56 ± 9.72	74.31 ± 12.31	<0.001
Systolic blood pressure, mmHg	98.88 ± 8.71	108.86 ± 10.72	<0.001	106.54 ± 11.52	112.03 ± 13.24	<0.001
Diastolic blood pressure, mmHg	59.70 ± 7.68	60.25 ± 8.15	0.127	64.45 ± 9.02	64.61 ± 10.02	0.668
Triglyceride, mg/dL	71.50 ± 30.18	72.96 ± 34.48	0.327	75.18 ± 33.46	75.40 ± 39.15	0.874
High-density lipoprotein, mg/dL	57.43 ± 12.54	52.02 ± 11.60	<0.001	58.32 ± 13.35	55.79 ± 13.51	<0.001
Fasting plasma glucose, mg/dL	93.41 ± 10.45	96.51 ± 8.78	<0.001	89.55 ± 8.71	92.29 ± 8.32	<0.001
IDF-adoMetS (SE), %	2.37 (0.49)	4.11 (0.64)	0.034	1.43 (0.32)	3.16 (0.48)	0.003
JIS-AdMetS (SE), %	3.30 (0.57)	4.53 (0.68)	0.163	2.72 (0.43)	3.46 (0.50)	0.262

IDF-adoMetS, International Diabetes Federation-defined adolescent MetS; JIS-AdMetS, Joint Interim Statement for adult MetS. ^1^ Distribution was displayed as mean ± standard deviation or percentage and standard error. ^2^ *p* values for sex difference were obtained adjusted for age, except for variable ‘age’.

**Table 2:** Factor loadings and characteristics for the first 3 principal components of obesity indicators in adolescents, stratified by sex, the ado-NAHSIT study.

Variables	Factor Loadings for Girls (*n* = 971)			Factor Loadings for Boys (*n* = 949)		
	PC1g	PC2g	PC3g	PC1b	PC2b	PC3b
**Adiposity indicators**						
Body mass index	0.419	−0.170	−0.004	0.414	−0.019	−0.193
Body adiposity index	0.399	−0.256	−0.062	0.383	−0.037	−0.165
Body roundness index	0.385	0.052	−0.012	0.366	−0.026	0.044
Waist-to-height ratio	0.387	0.056	−0.020	0.367	−0.025	0.046
Abdominal volume index	0.369	0.076	0.001	0.367	−0.003	−0.007
Waist circumference	0.366	0.100	−0.010	0.365	−0.005	0.003
A body shape index	−0.124	0.656	−0.019	−0.081	0.004	0.767
Conicity index	0.135	0.526	−0.027	0.200	−0.008	0.449
Waist-to-hip ratio	0.161	0.416	0.036	0.215	−0.025	0.377
Triglyceride-glucose index	−0.058	−0.048	0.650	−0.034	0.682	−0.004
Visceral adiposity index	−0.005	0.018	0.630	0.003	0.658	0.003
Lipid accumulation product	0.188	0.041	0.417	0.228	0.314	0.012
**Eigenvalue**	6.319	2.487	2.281	7.168	2.140	1.951
**Proportion of variance explained**	52.7%	20.7%	19.0%	59.7%	17.8%	16.3%
**Cumulative proportion**	52.7%	73.4%	92.4%	59.7%	77.6%	93.8%
**Factor characteristic of PC score**	Bodyfat-enhanced factor	Body-shape enhanced factor	Lipid-enhanced factor	Bodyfat-enhanced factor	Lipid-enhanced factor	Body-shape enhanced factor

PC1g-PC3g, principal components 1 to 3 for girls; PC1b-PC3b, principal components 1 to 3 for boys.

**Table 3:** Partial correlations of single and combined adiposity indicators with the number of abnormal metabolic syndrome components in adolescents, the ado-NAHSIT study.

	IDF-adoMetS				JIS-AdMetS			
Variables	Girls		Boys		Girls		Boys	
	pCorr ^1^	p*R*^2^	pCorr ^1^	p*R*^2^	pCorr ^1^	p*R*^2^	pCorr ^1^	p*R*^2^
**Single indicator**								
**Bodyfat-enhanced group**								
Body mass index	0.596 *	35.5%	0.618 *	38.2%	0.601 *	36.2%	0.618 *	38.2%
Body adiposity index	0.471 *	22.2%	0.525 *	27.6%	0.479 *	23.0%	0.525 *	27.6%
Body roundness index	0.606 *	36.7%	0.611 *	37.4%	0.607 *	36.9%	0.611 *	37.4%
Waist-to-height ratio	0.605 *	36.6%	0.601 *	36.2%	0.607 *	36.9%	0.601 *	36.2%
Abdominal volume index	0.613 *	37.6%	0.623 *	38.8%	0.611 *	37.3%	0.623 *	38.8%
Waist circumference	0.612 *	37.5%	0.610 *	37.2%	0.611 *	37.4%	0.610 *	37.2%
**Body-shape-enhanced group**								
A body shape index	0.022	0.1%	0.127 *	1.6%	0.006	0.0%	0.127 *	1.6%
Conicity index	0.376 *	14.1%	0.471 *	22.2%	0.364 *	13.3%	0.471 *	22.2%
Waist-to-hip ratio	0.443 *	19.6%	0.502 *	25.2%	0.437 *	19.1%	0.502 *	25.2%
**Lipid-enhanced group**								
Triglyceride-glucose index	0.402 *	16.2%	0.482 *	23.3%	0.414 *	17.1%	0.482 *	23.3%
Visceral adiposity index	0.544 *	29.6%	0.555 *	30.8%	0.565 *	31.9%	0.555 *	30.8%
Lipid accumulation product	0.613 *	37.6%	0.648 *	42.1%	0.606 *	36.7%	0.648 *	42.1%
**Combined indicator (score) ^2^**								
PC1	0.621 *	38.6%	0.622 *	38.7%	0.623 *	38.8%	0.622 *	38.7%
PC2	0.230 *	5.3%	0.558 *	31.1%	0.215 *	4.6%	0.558 *	31.1%
PC3	0.535 *	28.6%	0.254 *	6.5%	0.547 *	29.9%	0.254 *	6.5%

pCorr, partial correlation coefficient; p*R*^2^, partial *R*-square; PC, principal component; *, *p* < 0.05. ^1^ pCorr and p*R*^2^ were adjusted for study area, age, daily energy intake, physical activity, puberty status, cigarette smoking and alcohol drinking. ^2^ PC1, PC2, and PC3 were bodyweight-, bodyshape-, and lipid-enhanced factors, respectively, in girls. The corresponding PCs were bodyweight-, lipid-, and bodyshape-enhanced factors, respectively, in boys.

**Table 4:** Discriminations of single and combined adiposity indicators in the identification of adolescent metabolic syndrome in the ado-NAHSIT study.

	IDF-adoMetS										JIS-AdMetS									
	Girls					Boys					Girls					Boys				
Variables	AUC	Cutoff Point	Sen. %	Spe. %	YI	AUC	Cutoff Point	Sen. %	Spe. %	YI	AUC	Cutoff Point	Sen. %	Spe. %	YI	AUC	Cutoff Point	Sen. %	Spe. %	YI
**Single indicator**																				
**Bodyfat-enhanced group**																				
BMI	0.937 *	23.34	95.7	81.4	0.771	0.954*	27.10	97.4	89.0	0.864	0.913 *	23.34	93.8	82.1	0.759	0.925 *	27.10	88.4	89.0	0.773
BAI	0.841 *	29.94	91.3	65.4	0.567	0.904 *	27.39	97.4	75.8	0.733	0.830 *	31.12	81.3	76.4	0.576	0.876 *	27.39	93.0	75.9	0.690
BRI	0.924 *	3.60	95.7	82.4	0.780	0.943 *	3.60	100.0	82.7	0.827	0.907 *	3.60	93.8	83.1	0.768	0.912 *	3.60	93.0	82.8	0.758
WHtR	0.924 *	0.51	95.7	82.4	0.780	0.943 *	0.51	100.0	82.7	0.827	0.907 *	0.51	93.8	83.1	0.768	0.912 *	0.51	93.0	82.8	0.758
AVI	0.941 *	13.96	95.7	86.7	0.824	0.955 *	16.57	100.0	88.4	0.884	0.916 *	13.96	93.8	87.4	0.812	0.922 *	16.57	90.7	88.3	0.790
WC	0.941 *	82.7	95.7	86.3	0.819	0.955 *	90.5	100.0	88.2	0.882	0.916 *	82.7	93.8	87.0	0.808	0.922 *	90.5	90.7	88.2	0.789
**Body-shape-enhanced group**																				
ABSI	0.492	0.082	30.4	79.5	0.100	0.632 *	0.076	84.6	44.8	0.295	0.489	0.083	15.6	90.1	0.057	0.614 *	0.076	81.4	44.8	0.262
CoI	0.767 *	1.26	52.2	89.8	0.419	0.896 *	1.22	92.3	83.1	0.754	0.758 *	1.26	53.1	90.2	0.433	0.864*	1.22	86.0	83.1	0.692
WHR	0.826 *	0.82	82.6	70.6	0.532	0.898 *	0.85	92.3	80.0	0.723	0.833 *	0.82	81.3	71.0	0.523	0.871 *	0.85	88.4	80.1	0.685
**Lipid-enhanced group**																				
TGI	0.849 *	8.55	73.9	92.4	0.663	0.860 *	8.24	87.2	70.4	0.576	0.853 *	8.55	71.9	93.0	0.648	0.872 *	8.40	79.1	80.7	0.598
VAI	0.915 *	4.21	73.9	92.7	0.666	0.877 *	2.60	76.9	82.6	0.596	0.910 *	3.60	78.1	88.4	0.665	0.887 *	2.60	79.1	83.0	0.621
LAP	0.942 *	91.67	95.7	79.3	0.750	0.956 *	93.33	100.0	82.0	0.820	0.921 *	137.87	78.1	93.6	0.717	0.938 *	93.33	97.7	82.2	0.799
**Combined indicator ^1^**																				
PC1 score	0.939 *	1.61	95.7	81.9	0.775	0.953 *	2.54	100.0	86.4	0.864	0.918 *	2.69	87.5	89.8	0.773	0.922 *	2.54	93.0	86.4	0.794
PC2 score	0.669 *	1.56	52.2	85.9	0.380	0.883 *	0.81	79.5	80.8	0.603	0.673 *	1.06	56.3	78.5	0.347	0.893 *	0.81	81.4	81.1	0.625
PC3 score	0.887 *	1.97	73.9	93.7	0.676	0.732 *	0.23	79.5	62.5	0.420	0.887 *	1.42	78.1	90.2	0.683	0.710 *	0.23	74.4	62.5	0.369

IDF-adoMetS, International Diabetes Federation-defined adolescent MetS; JIS-AdMetS, Joint Interim Statement for adult MetS; AUC, area under receiver operating characteristic curve; Sen., sensitivity; Spe., specificity; YI, Youden’s index; BMI, body mass index; BAI, body adiposity index; BRI, body roundness index; WHtR, waist-to-height ratio; AVI, abdominal volume index; WC, waist circumference; ABSI, a body shape index; CoI, conicity index; WHR, waist-to-hip ratio; TGI, triglyceride-glucose index; VAI, visceral adiposity index; LAP, lipid accumulation product; PC, principal component. * denotes *p* < 0.05 for a significant discriminatory ability of adiposity indicator using AUC analysis. ^1^ PC1, PC2, and PC3 were bodyweight-, bodyshape-, and lipid-weighted factors, respectively, in girls, and the corresponding PCs were bodyweight-, lipid-, and bodyshape-weighted factors in boys.

**Table 5:** Discriminations of selected adiposity indicators in the identification of adolescent metabolic syndrome in the validation data in the mRP-aMS study.

	Girls (*n* = 1399)										Boys (*n* = 1328)									
Factors	IDF-adoMetS Proportion = 1.43%					JIS-AdMetS Proportion = 2.72%					IDF-adoMetS Proportion = 3.16%					JIS-AdMetS Proportion = 3.46%				
	AVI	WC	WHR	LAP	PC1	AVI	WC	WHR	LAP	PC1	AVI	WC	WHR	LAP	PC1	AVI	WC	WHR	LAP	PC1
**Cutoff point**	13.96	82.7	0.82	91.67	1.61	13.96	82.7	0.82	137.87	2.69	16.57	90.5	0.85	93.33	2.54	16.57	90.5	0.85	93.33	2.54
**Discrimination**																				
Sensitivity (SE), %	90.0	90.0	85.0	85.0	95.0	81.6	81.6	84.2	76.3	92.1	95.2	97.6	95.2	92.9	97.6	87.0	89.1	89.1	93.5	91.3
	(6.7)	(6.7)	(8.0)	(8.0)	(4.9)	(6.3)	(6.3)	(5.9)	(6.9)	(4.4)	(3.3)	(2.4)	(3.3)	(4.0)	(2.4)	(5.0)	(4.6)	(4.6)	(3.6)	(4.2)
Specificity (SE), %	91.6	91.4	79.8	85.7	82.2	92.4	92.3	80.7	94.1	90.4	90.7	90.7	75.9	83.4	87.4	90.7	90.7	75.9	83.7	87.4
	(0.7)	(0.8)	(1.1)	(0.9)	(1.0)	(0.7)	(0.7)	(1.1)	(0.6)	(0.8)	(0.8)	(0.8)	(1.2)	(1.0)	(0.9)	(0.8)	(0.8)	(1.2)	(1.0)	(0.9)
Youden’s index	0.816	0.814	0.648	0.707	0.772	0.740	0.739	0.649	0.704	0.826	0.860	0.884	0.711	0.763	0.850	0.777	0.798	0.650	0.772	0.787
No. of positive test per case identified	7.4	7.6	17.4	12.6	13.9	4.3	4.4	9.2	3.8	4.7	4.0	3.9	8.8	6.5	5.0	4.0	3.9	8.5	5.9	4.8
Total no. of test per case identified	77.7	77.7	82.3	82.3	73.6	45.1	45.1	43.7	48.2	40.0	33.2	32.4	33.2	34.1	32.4	33.2	32.4	32.4	30.9	31.6

IDF-adoMetS, International Diabetes Federation-defined adolescent MetS; JIS-AdMetS, Joint Interim Statement for adult MetS; AVI, abdominal volume index; WC, waist circumference; WHR, waist-to-hip ratio; LAP, lipid accumulation product; PC1, bodyfat-enhanced principal component; SE, standard error.

## Supplementary Materials

The following are available online at https://www.mdpi.com/2072-6643/12/10/3165/s1, Table S1: Criteria of metabolic syndrome (MetS) and its abnormal components (AC) defined by IDF and JIS-Adult.

## Abbreviations

ABSI: a body shape index; ado-NAHSIT, nutrition and health survey in Taiwan for adolescents; AUC, area under receiver operating characteristic curve; VI, abdominal volume index; BAI, body adiposity index; BMI, body mass index; BRI, body roundness index; CoI, conicity index; IDF-adoMetS, the International Diabetes Federation-defined criteria for adolescent MetS; JIS-AdMetS, the Joint Interim Statement for adult MetS; LAP, lipid accumulation product; MetS, metabolic syndrome; mRP-aMS, multilevel risk profiles for adolescent metabolic syndrome; OR, odds ratio; PC, principal component; pCorr, partial correlations; p*R*^2^, partial *R*-square; TGI, triglyceride-glucose index; VAI, visceral adiposity index; WC, waist circumference; WHtR, waist-to-height ratio; WHR, waist-to-hip ratio; YI, Youden’s index.
