# Supplementary material for: Characteristic-Grouped Adiposity Indicators for Identifying Metabolic Syndrome in Adolescents: Develop and Valid Risk Screening Tools Using Dual Population
Source: Nutrients. 2020 Oct 16;12(10):3165. doi: 10.3390/nu12103165 (PMC7602786; doi:10.3390/nu12103165)
Supplement: Supplementary file 1 [file nutrients-12-03165-s001.pdf]

Table S1. Criteria of metabolic syndrome (MetS) and its abnormal components (AC) defined by IDF and JIS-Adult.

MetS components.	Central obesity	High blood pressure	Low HDL-C	Increased triglyceride	Elevated fasting glucose	Diagnostic criteria of MetS
**IDF-Adolescent** [32]						
**10-15 years**	WC ≥90 percentile (or adult cutoff if lower)[1,2]	SBP ≥130 / DBP ≥85 mmHg (or antihypertensive drug treatment)	<40 mg/dL	≥150 mg/dL	≥100 mg/dL (or previously diagnosed type 2 diabetes)	**Central obesity + 2 other ACs**
**16-18 years**	WC ≥90 percentile (or adult cutoff if lower)[1,2]	SBP ≥130 / DBP ≥85 mmHg (or antihypertensive drug treatment)	<40 mg/dL in males, <50 mg/dL in females	≥150 mg/dL	≥100 mg/dL (or previously diagnosed type 2 diabetes)	**Central obesity + 2 other ACs**
**JIS-Adult (for Asians ≥18 years)** [20]	WC ≥90 cm in males, WC ≥80 cm in females[3]	SBP ≥130 / DBP ≥85 mmHg (or antihypertensive drug treatment)	<40 mg/dL in males, <50 mg/dL in females	≥150 mg/dL	≥100 mg/dL (or previously diagnosed type 2 diabetes)	**Any 3 or more ACs**
**Difference in definition**[4]	**No**	**No**	**Yes**	**No**	**No**	**Yes**

Abbreviations: IDF-Adolescent, International Diabetes Federation definition of MetS for adolescents; JIS-Adult, Joint Interim Statement of MetS for adults; WC, waist circumference; HDL-C, high-density lipoprotein cholesterol; SBP, systolic blood pressure; DBP, diastolic blood pressure. [1] The 90 percentiles of WC in this nationwide data were both higher than that of Asian adult cutoff points, therefore, the adult cutoff points (i.e., WC ≥90 cm in males and ≥80 cm in females) were applied. [2]Central obesity can be assumed if BMI is >30 kg/m^2, and WC does not need to be considered. [3]The recommended WC thresholds of abdominal obesity for Asian adults (i.e., WC ≥90 cm in males and ≥80 cm in females) were applied. [4]This denotes whether there were differences in the diagnosis of MetS and its components across 3 criteria.
